# Cytokines and microbicidal molecules regulated by IL-32 in THP-1-derived human macrophages infected with New World *Leishmania* species

**DOI:** 10.1371/journal.pntd.0005413

**Published:** 2017-02-27

**Authors:** Jéssica Cristina dos Santos, Bas Heinhuis, Rodrigo Saar Gomes, Michelle S. M. A. Damen, Fernando Real, Renato A. Mortara, Samuel T. Keating, Charles A. Dinarello, Leo A. B. Joosten, Fátima Ribeiro-Dias

**Affiliations:** 1 Department of Internal Medicine and Radboud Center of Infectious Diseases (RCI), Radboud University Medical Center, Nijmegen, The Netherlands; 2 Instituto de Patologia Tropical e Saúde Pública, Universidade Federal de Goiás, Goiânia, Brazil; 3 Departamento de Microbiologia, Imunologia e Parasitologia, Escola Paulista de Medicina, Universidade Federal de São Paulo, Brazil; 4 School of Medicine, Division of infectious diseases, University of Colorado Denver, Aurora, Colorado, United States of America; Universidade Federal de Minas Gerais, BRAZIL

## Abstract

**Background:**

Interleukin-32 (IL-32) is expressed in lesions of patients with American Tegumentary Leishmaniasis (ATL), but its precise role in the disease remains unknown.

**Methodology/Principal findings:**

In the present study, silencing and overexpression of IL-32 was performed in THP-1-derived macrophages infected with *Leishmania* (*Viannia*) *braziliensis or L*. (*Leishmania*) *amazonensis* to investigate the role of IL-32 in infection. We report that *Leishmania* species induces IL-32γ, and show that intracellular IL-32γ protein production is dependent on endogenous TNFα. Silencing or overexpression of IL-32 demonstrated that this cytokine is closely related to TNFα and IL-8. Remarkably, the infection index was augmented in the absence of IL-32 and decreased in cells overexpressing this cytokine. Mechanistically, these effects can be explained by nitric oxide cathelicidin and β-defensin 2 production regulated by IL-32.

**Conclusions:**

Thus, endogenous IL-32 is a crucial cytokine involved in the host defense against *Leishmania* parasites.

## Author summary

*Leishmania* (*V*.) *braziliensis* and *L*. (*L*.) *amazonensis* are protozoa that infect macrophages and cause cutaneous and mucosal leishmaniasis. Here we showed that both *Leishmania* species induce the production of IL-32γ in human macrophages. This intracellular and pro-inflammatory cytokine mediates the production of cytokines, especially TNFα and IL-8, in *Leishmania*-infected macrophages. Differential effects of IL-32γ on TNFα, IL-10 and IL-1Ra production after infection with distinct *Leishmania* species were detected, consistent with the concept that IL-32γ can differently influence the outcome of inflammatory process in leishmaniasis lesions. Moreover, IL-32γ upregulates microbicidal molecules, antimicrobial peptides, as well as NO, which are known as important factors in parasite control. These results underscore IL-32γ as a crucial cytokine to host defense against leishmaniasis.

## Introduction

Interleukin-32 (IL-32) is a predominantly intracellular proinflammatory cytokine [1] that can be expressed in nine different isoforms (IL-32α, IL-32β, IL-32γ, IL-32δ, IL-32ε, IL-32ζ, IL-32η, IL-32θ and IL-32σ) [2]. This cytokine can induce production of tumor necrosis factor alpha (TNFα), IL-8, IL-6, and IL-1β in THP-1 and RAW264.7 macrophages cell lines [3,4], with IL-32γ being the most active isoform [5].

Induction of IL-32α and IL-32γ during *Mycobacterium tuberculosis* (MTB) infection mediates TNFα, IL-6, IL-1β production and macrophage apoptosis that is involved in protection against MTB [6,7]. In addition, IL-32/vitamin D/antimicrobial peptides axis control MTB infection [8]. IL-32 is associated with strong Th1 immune response, controlling *M*. *leprae* infection [9]. In viral infections, induction of IL-32 is associated with the control of viral replication [10–12], but also with inflammation and tissue lesion [13–16]. In protozoan infections, IL-32 has been identified in lesions of patients with American Tegumentary Leishmaniasis (ATL) [17].

ATL is a vector-borne disease caused by *Leishmania* parasites. In general, *L*. (*Viannia*) *braziliensis* cause localized cutaneous (LCL) and oral/ nasal mucosal lesions (ML). LCL can cure spontaneously or after treatment. By contrast ML does not spontaneously heal and recurrence is frequent after treatment. In addition to these clinical forms, *L*. (*Leishmania*) *amazonensis* can cause diffuse cutaneous leishmaniasis (DCL), which it is not cured even after treatment [18–20]. A moderate or strong Th1 response is present in infections caused by *L*. (*V*.) *braziliensis* whereas patients infected with *L*. (*L*.) *amazonensis* present a less potent Th1-type response or can be anergic [21]. The strong Th1-type immune response is important for controlling the infection but also causes inflammation and pathology [22,23]. Th1-type cytokines (IFNγ and TNFα) activate infected monocytes or macrophages to secrete microbicidal molecules such as oxygen and nitrogen reactive species, which are crucial for the parasite killing [24–28]. During *Leishmania* infection, macrophages can produce proinflammatory cytokines (TNFα, IL-1β, IL-8) and regulatory (IL-10, IL-1Ra) molecules [29–31]. Thus a balance between pro- and anti-inflammatory mediators during the immune responses is critical to control inflammatory diseases [32,33].

The mechanisms responsible for persistence of the parasite and immunopathology of leishmaniasis remain unclear. We previously reported that IL-32γ is expressed in cutaneous and mucosal lesions of patients with ATL caused by *Leishmania* (*Viannia*) species and also that *L*. (*V*.) *braziliensis* induces IL-32γ in peripheral blood mononuclear cells (PBMC) [17]. Here, we investigated whether distinct isoforms of IL-32 can be induced by *L*. (*V*.) *braziliensis* and *L*. (*L*.) *amazonensis*, and whether IL-32 can regulate cytokine and microbicidal activity of human macrophages infected with these two New World *Leishmania* species.

## Methods

### Ethics statement

The study used only cell lines and parasites. The whole project was approved by Ethical Commitee of Hospital das Clínicas/Universidade Federal de Goiás, Brazil, prot. n. 44033514.0.0000.5078.

### THP-1 cell line and *Leishmania* cultures

THP-1 cell line was obtained from ATCC (Manassas, VA). Cells were cultured in RPMI-1640 medium (Gibco—Life Technologies) supplemented with 10% heat-inactivated fetal bovine serum (FBS; Gibco—Life Technologies), 10 mM of pyruvate, 10 mM L-glutamine, 100 U/mL of penicillin and 100 μg/mL streptomycin (Sigma—Aldrich).

*L*. (*L*.) *amazonensis* (IFLA/BR/67/PH8) reference strain and MHOM/BR/2003/IMG *L*. (*V*.) *braziliensis*, a clinical isolate obtained from cutaneous lesion of LCL patient (Leishbank IPTSP/UFG) [34], were used. Promastigotes forms were cultured in Grace’s Insect Medium, (Gibco—Life Technologies) supplemented with heat-inactivated 20% FBS (Sigma—Aldrich) and 100 U/mL of penicillin/streptomycin (Sigma—Aldrich) at 26°C. Parasites of *L*. *(L*.*) amazonensis* from stationary phase (6^th^—7^th^ day) of growth were used to infect macrophages derived from THP-1 cells. For *L*. *(V*.*) braziliensis*, parasites were collected from stationary phase (6^th^—7^th^ day) of growth and metacyclic promastigotes were negatively selected using *Bauhinia purpurea* lectin according to the protocol described by [35]. Parasites were washed three times with sterile phosphate-buffered saline (PBS) pH 7.4 (1,000 g, 10 min, 10°C). The suspensions were diluted into 0.4% formaldehyde in PBS for parasite quantification by hemocytometer.

### THP-1-derived macrophages and stimulation

A previously described protocol [36] was used for THP-1 cell differentiation into macrophages with some alterations. According to the type of experiment, cell numbers were adapted to cultures with or without cover slides. Briefly, cells were cultured with phorbol myristate acetate (PMA, Sigma-Aldrich) at 100 ng/mL. After 48 h (37°C, 5% CO2), cells were gently washed with warm medium and incubated for an additional 48 h. Medium was replenished and cells were incubated for a further 24 h. Parasites (at multiplicity of infection (MOI) ~ 5:1) of both *Leishmania* species were added into the THP-1-derived macrophage cultures and 100 ng/mL of *E. coli* LPS (O111:B4 Sigma-Aldrich) was used as a control. This commercial LPS was further purified based on [37]. In some experiments, THP-1-derived macrophages were preincubated for 1 h in the absence or presence of neutralizing antibodies to TNFα (5 μg/mL, Adalimumab) or IgG control (5 μg/mL). After 4 h, non-internalized parasites were washed out, medium was replaced and cultures were incubated for indicated times.

### Confocal microscopy

After THP-1-derived macrophage infection (2 x 10^5^ cells/0.5 mL, grown over coverslips in 24-wells plates; MOI: 5:1; 24 h), cells were fixed with 4% paraformaldehyde and blocked/permeabilized with 0.1% saponin, 10% FBS, 5% goat serum and 5% human serum solution (block solution). Primary antibodies to IL-32 (rabbit polyclonal antibody 5 μg/mL; Abcam;) and to LAMP1 (mouse H4B4, IgG1; 1/2 culture supernatants; to identify lysosomal proteins and parasitophorous vacuoles); secondary antibodies—Alexa Fluor 594 goat anti-rabbit IgG (H+L), 1/200, to detect IL-32 (Molecular Probes) and anti-mouse IgG (whole molecule) F(ab)´_2_ fragment of sheep antibody-Cy3 conjugate, 1/200, to detect LAMP2 (Sigma-Aldrich); and control antibodies were all in block solution. A solution of 4´,6-diamidino-2-phenylindole (DAPI; 10 μg/mL; Invitrogen, Life Technologies) was used to stain nucleus/DNA, and fluorescent mounting medium (Dako) was used to prepare the coverslips for confocal microscopy. Images were acquired in a Leica TCS SP5 II confocal microscope.

### Silencing and overexpression of IL-32 by siRNA or plasmid

THP-1 cells (15 x 10^6^ cells/15 mL) were differentiated into macrophages (75 cm^2^—tissue culture flask; Corner) for 5 days, as described above. 2.5 x 10^6^ cells/800 μL were electroporated by using Amaxa Nucleofector technology (Lonza, Basel) according with the protocol described in reference [38]. For knockdown of IL-32, 1 μg of ON-TARGETplus SMARTpool siRNA (S1 Table) per transfection was used or 1 μg of ON-TARGETplus SMARTpool control siRNA (Dharmacon Inc). For IL-32 overexpression, 0.5 μg of pCDNA3 plasmid expressing human IL-32γ or egfp was used as a control. Transfected cells (3 x 10^5^/100 μL) were plated on to flat-bottom 96-well plates (Costar) with or without 6 mm coverslips and 100 μL of transfection medium were added. Twenty-four hours post-transfection, the medium was replaced and 1.5 x 10^6^ parasites of either *Leishmania* species were added to the cultures. After 4 h and 24 h, supernatants were collected and stored at -20°C until cytokine measurement; after 4 h, 24 h or 48 h the cell monolayers were collected by adding 200 μL of TRIzol and stored at -80°C until mRNA extraction. After incubation, coverslips were collected to measure macrophage infection index. We performed comparable experiments to determine transfection (egfp) efficiency and this was around 30%. It is important to notice that according to the protocol used for silencing and overexpression [38], the general protocol described above needed alterations. After derivation with PMA, cells were transfected and they rested 24 h in medium containing 5% and 20% of human serum for silencing and overexpression, respectively. In addition, to keep cells adhered additional PMA was added to the cultures (2.5 ng/mL). Thus, the results are comparable among them only in each set of experiments in the same conditions (WT cells vs transfected cells).

### mRNA expression by quantitative real-time PCR (qPCR)

RNA isolation was carried out based on the method reported by [39]. RNA was precipitated with isopropanol and washed with 75% ethanol followed by reconstitution in RNAse-free water. Subsequently, RNA was reverse transcribed into cDNA by using iScript (Bio-Rad, Hercules, CA, USA). Diluted cDNA was used for qPCR analysis that was done by using the StepOnePlus sequence detection systems (Applied Biosystems, Foster City, CA, USA) with SYBR Green Mastermix (Applied Biosystems). Primer sequences (S2 Table) for IL-32 were previously developed by [3,40] whereas other primer sequences (TNFα, IL-1β, IL-8, IL-1Ra, IL-10, inducible nitric oxide synthase [iNOS], cathelicidin, b-defensin) were obtained from Harvard Primerbank database. Primers were purchased from Biolegio. The mRNA analysis was done with the 2^dCt x 1000 method and normalized against the housekeeping gene GAPDH.

### Assessment of *Leishmania*-induced mediators

Human TNFα, IL-8, IL-1β, IL1-Ra, IL-10 and LL-37 (cathelicidin) were determined in culture supernatants using commercial Enzyme-Linked Immunosorbent Assay (ELISA) kits (Sanquin, R&D Systems and Hycult biotech). Intracellular IL-32 protein was measured in cell lysates collected with Triton-X100 by using an IL-32 ELISA (R&D Systems). Nitric Oxide (NO) production was determined in culture supernatants with Griess reagent to detect nitrite (Sigma-Aldrich). Cell death was monitored by measuring the release of lactate dehydrogenase (LDH) in the supernatants by using a Cytotox 96 kit (Promega).

### Evaluation of macrophage infection

After incubation, the coverslips were collected, fixed and stained with Giemsa (Merck Millipore) and analyzed under a light microscope (1000x) to determine the infection index. Three hundred cells were analysed and the percentage of infected cells and the mean number of intracellular parasites per infected cell were determined. Infection index = percentage of infected cells × mean number of parasites per infected cell.

### Statistical analysis

Data represent mean ± SEM (standard error of the mean). All data were evaluated by OneWay ANOVA/Bonferroni test using GraphPad Prism v.6 software (San Diego, CA, USA). Level of significance was established at p < 0.05.

## Results

### *Leishmania*-induced intracellular IL-32γ production is dependent on TNFα

**Fig 1:**
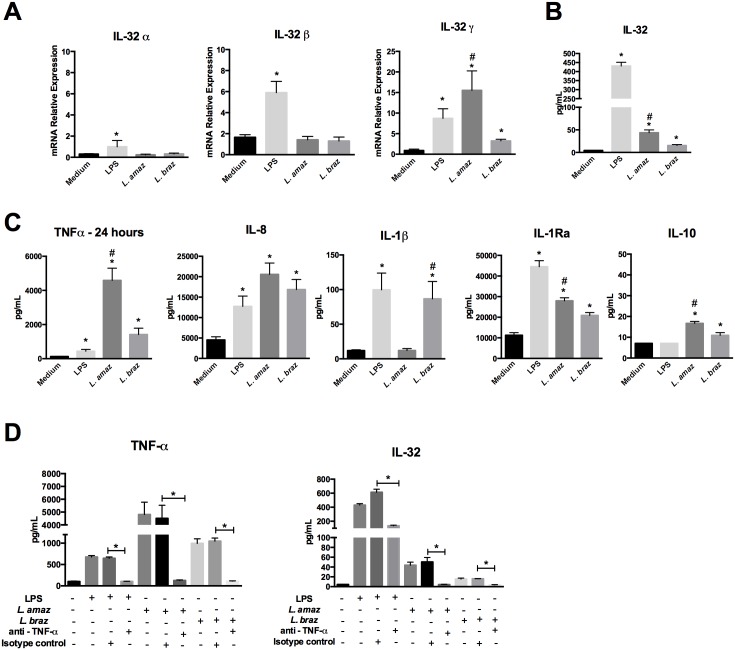
*Leishmania* induces IL-32γ expression in a TNFα-dependent manner. PMA-differentiated THP-1 cells (1 x 10^6^ cells/mL) were infected with promastigote forms (5 x 10^6^ parasites) in the growth stationary phase of *L*. (*L*.) *amazonensis* (*L*. *amaz*), metacyclic promastigote forms (5 x 10^6^ parasites) of *L*. (*V*.) *braziliensis* (*L*. *braz*) or LPS (100 ng/mL) as a positive control during 4 h. Cells were washed to remove non-internalized parasites and incubated for 24 h. (A) mRNA expression of isoforms α, β and γ of IL-32 was determined by quantitative real-time PCR. (B) Intracellular IL-32 protein levels were determined by ELISA in cell lysates. (C) TNFα, IL-8, IL-1β, IL-1Ra, IL-10 productions were determined by ELISA in culture supernatants. (D) Antibodies to TNFα (anti-TNFα, 5 μg/ml) or isotype control (5 μg/mL) were added 30 min before addition of LPS (100 ng/mL) or *Leishmania* sp. TNFα levels (left panel) and intracellular IL-32 (right panel) were determined by ELISA in supernatants and cell lysates, respectively. Values are expressed as means ± SEM of three independent experiments. *p < 0.05 (Medium vs LPS, *L*. *amaz*, *L*. *braz*); #p < 0.05 (*L*. *amaz*, *L*. *braz*).

We detected a significant induction of IL-32γ, but not IL-32β or IL-32α for both *L*. (*L*.) *amazonensis* or *L*. (*V*.) *braziliensis* infection (24 h; Fig 1A). *L*. (*L*.) *amazonensis* induced higher IL-32γ expression than *L*. (*V*.) *braziliensis* (Fig 1A). IL-32γ time course showed that IL-32γ mRNA started to increase after 4 h and achieved a peak at 24 h (S1A Fig—left panel). It is known that IL-32γ mRNA can be spliced into IL-32β and IL-32α [3]. Here we showed that in THP-1 cultures *Leishmania*, in contrast to LPS, only induced IL-32γ (Fig 1A and S1A Fig—right panel). The intracellular IL-32 protein levels (Fig 1B), paralleling changes in mRNA expression, were higher in *L*. (*L*.) *amazonensis* than in *L*. (*V*.) *braziliensis*-infected macrophages.

Both *L*. (*L*.) *amazonensis* and *L*. (*V*.) *braziliensis* induced significant amounts of TNFα, IL-8, IL-1Ra and IL-10. Time course production of these cytokines showed that 24 h was the best cutoff point to establish relationship between these cytokines and IL-32 production (S2 Fig). TNFα levels were increased after 24 h of infection with both species (Fig 1C) and were higher than TNFα produced after 4 h, whereas LPS–induced TNFα production declined from 4 h to 24 h (S1B Fig). Remarkably, only *L*. (*V*.) *braziliensis* induced a major increase in IL-1β production. *L*. (*L*.) *amazonensis* induced considerably higher levels of TNFα, IL-1Ra and IL-10 than *L*. (*V*.) *braziliensis* (Fig 1C).

Since *Leishmania*-induced IL-32 and TNFα showed similar time course production (increase from 4 h to 24 h), to explore the influence of *Leishmania*-induced TNFα in the production of IL-32, TNFα was efficiently blocked using specific antibodies during *Leishmania* species infection (Fig 1D, left panel), leading to a significant reduction in intracellular IL-32 production (Fig 1D, right panel). Comparable results were obtained when LPS was used to induce TNFα (Fig 1D, left panel) and IL-32 (Fig 1D, right panel). Increased intracellular IL-32γ concentrations are associated with cell death [41]. However we observed LDH concentrations remained stable following *Leishmania* infection (S1C Fig).

**Fig 2:**
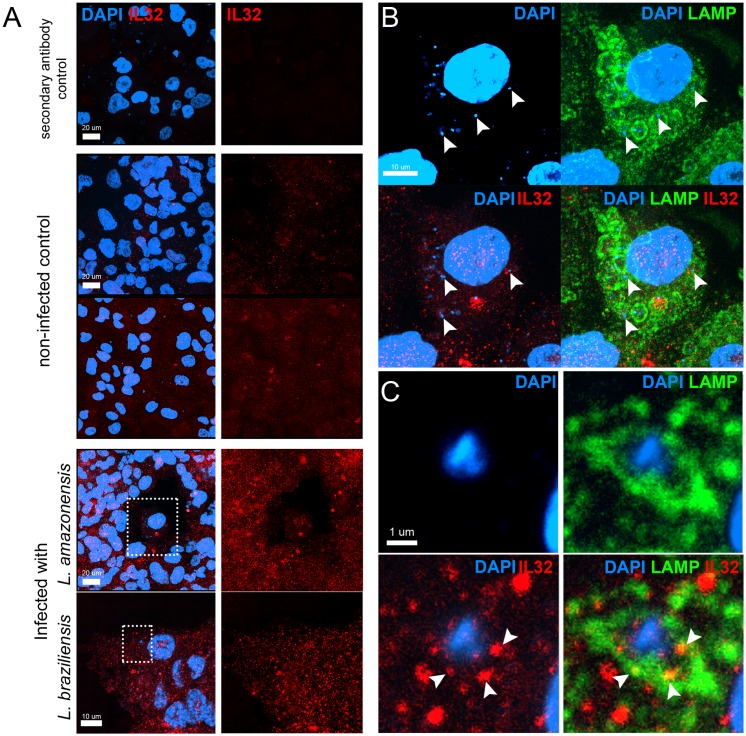
Intracellular distribution of IL-32 after *Leishmania* species infection. PMA-differentiated THP-1 cells (2 x 10^5^ cells/0.5 mL) were infected with promastigote forms (10 x 10^5^ parasites) in stationary phase of growth of *L*. (*L*.) *amazonensis* or metacyclic promastigote forms (10 x 10^5^ parasites) of *L*. (*V*.) *braziliensis* during 4 h. Afterwards, cells were washed to remove non-internalized parasites and incubated for 24 h. Cells were stained for IL-32 (rabbit polyclonal antibody; red), lysosomal-associated membrane protein, LAMP1 (mouse monoclonal antibody; green) and dapi (blue) for confocal microscopy. Yellow colour indicates colocalization of IL-32 and LAMP1. (A) In the eight first images, bar = 20 μm; two inferior images, bar = 10 μm (*L*. (*V*.) *braziliensis*). (B) *L*. (*L*.) *amazonensis*, white arrow heads indicate parasites present in LAMP1^+^-parasitophorous vacuoles; bar = 10 μm; (C) *L*. *(V*.*) braziliensis*, white arrow heads indicate colocalization of IL-32 and LAMP1 in a LAMP1^+^-parasitophorous vacuole containing one amastigote (blue), bar = 1 μm; images were took from (A) (left bottom, dashed white squares).

Next we investigated the cellular distribution of IL-32 after *Leishmania* species infection. In Fig 2A is depicted general staining for IL-32 in uninfected or *Leishmania*-infected cells. As showed in Fig 2B IL-32 localizes to both the cytoplasm and nucleus of macrophages. In some preparations, IL-32 co-localized with lysosomes, however co-localization with *Leishmania* containing parasitophorous vacuoles was rare (Fig 2C).

### Silencing or overexpression shows that IL-32γ regulates cytokine production induced by *Leishmania* species

**Fig 3:**
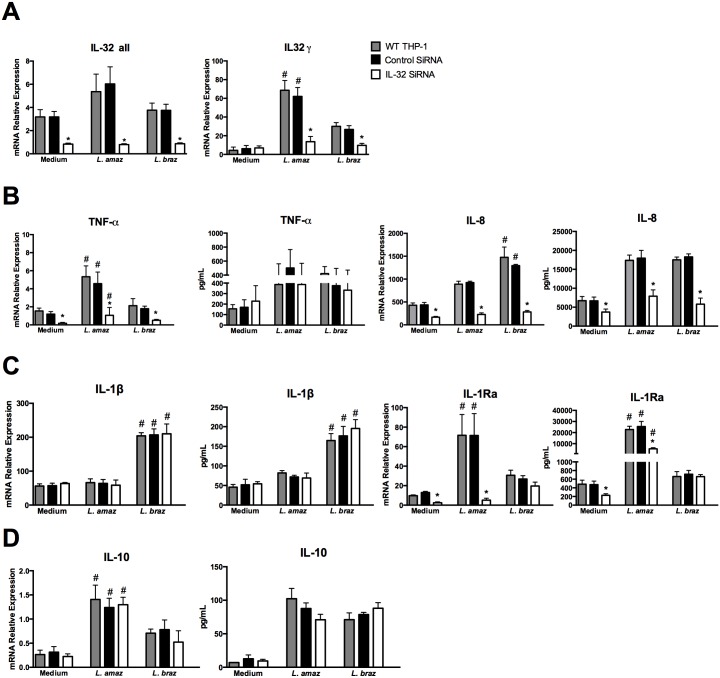
Decrease of cytokine production after IL-32 silencing in THP-1-derived macrophages infected with *Leishmania* species. PMA-differentiated THP-1 cells (2.5 x 10^6^ cells/800 μL) were electroporated by using Amaxa Nucleofector Technology with IL-32 siRNA (for IL-32 knockdown) and control siRNA according to the protocol described in [38]. The final concentration per well was 3 x 10^5^ cells/100 μL. After 24 h of transfection, cells were infected with promastigote forms (1.5 x 10^6^ parasites) in the growth stationary phase of *L*. (*L*.) *amazonensis* (*L*. *amaz*) or metacyclic promastigote forms (1.5 x 10^6^ parasites) of *L*. (*V*.) *braziliensis* (*L*. *braz*). After 4 h, non-internalized parasites were washed out and cells were incubated for 24 h. (A) mRNA expression of IL-32γ isoform and all isoforms were determined by quantitative real-time PCR. mRNA expression and protein levels of (B) TNFα and IL-8, (C) IL-1β and IL-1Ra and (D) IL-10 were determined by quantitative real-time PCR and ELISA in supernatants, respectively. Values are expressed as means ± SEM of three independent experiments. *p < 0.05 (Control SiRNA vs IL-32 SiRNA); #p < 0.05 (*L*. *amaz*, *L*. *braz*).

We investigated whether endogenous IL-32 is directly involved in the enhanced production of pro- and anti-inflammatory cytokines observed after *Leishmania* infection. To verify whether the transfection procedure may interfere in the capacity of THP-1-derived macrophages to produce cytokines, cells without transfection (WT THP-1) were also investigated. IL-32 mRNA expression (all IL-32 isoforms and IL-32γ) was silenced by RNA interference (Fig 3A). Silencing of endogenous IL-32γ decreased TNFα mRNA expression at 24 h (p < 0.05), but not TNFα protein levels (Fig 3B). In addition, after 4 h neither the TNFα mRNA nor the protein was significantly affected by silencing of IL-32 (S3A Fig). Both IL-8 mRNA and IL-8 protein were strongly reduced after infection with either *Leishmania* species when IL-32 was silenced (Fig 3B). Interestingly, when IL-32 expression was silenced, the levels of induced IL-1β and IL-1Ra mRNA and protein expression induced by *L*. (*V*.) *braziliensis* were not altered (Fig 3C). By contrast, IL-1Ra mRNA and IL-1Ra protein were decreased after infection with *L*. (*L*.) *amazonensis* and silencing of IL-32 (Fig 3C). No differences were observed for IL-10 mRNA expression and protein production for both *Leishmania* species in IL-32 knockdown cells (Fig 3D).

**Fig 4:**
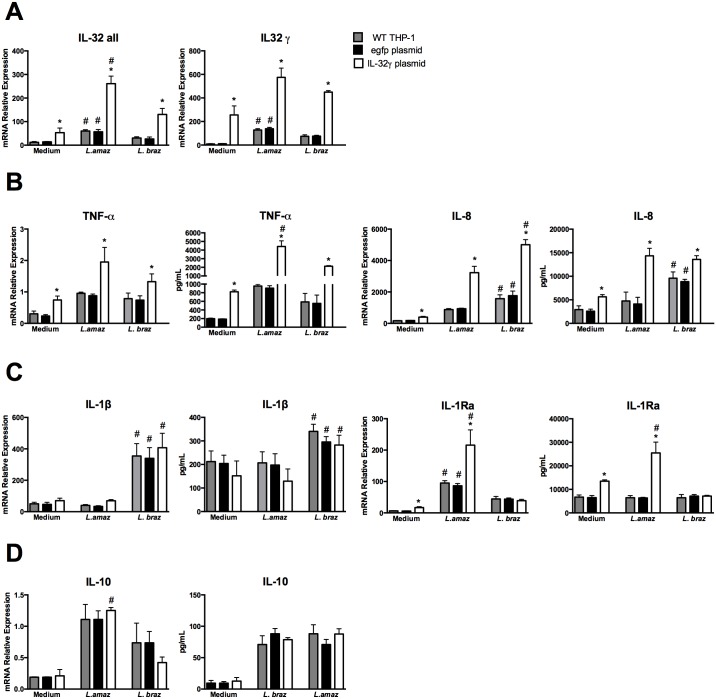
Increased cytokine production after overexpression of IL-32 in human THP-1-derived macrophages infected with *Leishmania* species. PMA-differentiated THP-1 cells (2.5 x 10^6^ cells/800 μL) were electroporated by using Amaxa Nucleofector Technology with IL-32 plasmid (for IL-32 overexpression) and egfp plasmid (as a control) according to the protocol described in [38]. The final concentration per well was 3 x 10^5^ cells/100 μL. After 24 h of transfection, cells were infected with promastigote forms (1.5 x 10^6^ parasites) in the growth stationary phase of *L*. (*L*.) *amazonensis* (*L*. *amaz*), metacyclic promastigote forms (1.5 x 10^6^ parasites) of *L*. (*V*.) *braziliensis* (*L*. *braz*). After 4 h cells were washed and incubated for 24 h. (A) mRNA expression of IL-32γ isoform and all IL-32 isoforms was determined by quantitative real-time PCR. mRNA expression and protein levels of (B) TNFα and IL-8, (C) IL-1β and IL-1Ra and (D) IL-10 were determined by quantitative real-time PCR and ELISA in supernatants, respectively. Values are expressed as means ± SEM of three independent experiments. *p < 0.05 (egpf plasmid vs IL-32γ plasmid); #p < 0.05 (*L*. *amaz*, *L*. *braz*).

Overexpression of IL-32γ (Fig 4A) resulted in significant increases in TNFα and IL-8 mRNA expression and protein production after infection with either *Leishmania* species in comparison with egfp transfected control cells (Fig 4B). The presence of high levels of IL-32γ caused an impressive increase of TNFα mRNA fast after 4 h of incubation without significant alteration in protein levels (S3C Fig). In accordance with IL-32γ silencing results, no differences in IL-1β levels were observed whereas increased IL-1Ra mRNA and protein expression was detected only after infection with *L*. (*L*.) *amazonensis* (Fig 4C). No differences were found between the two *Leishmania* species on IL-10 mRNA expression and proteins levels (Fig 4D).

### IL-32γ regulates nitric oxide and cathelicidin production, microbicidal molecules that can control *Leishmania* species infection

**Fig 5:**
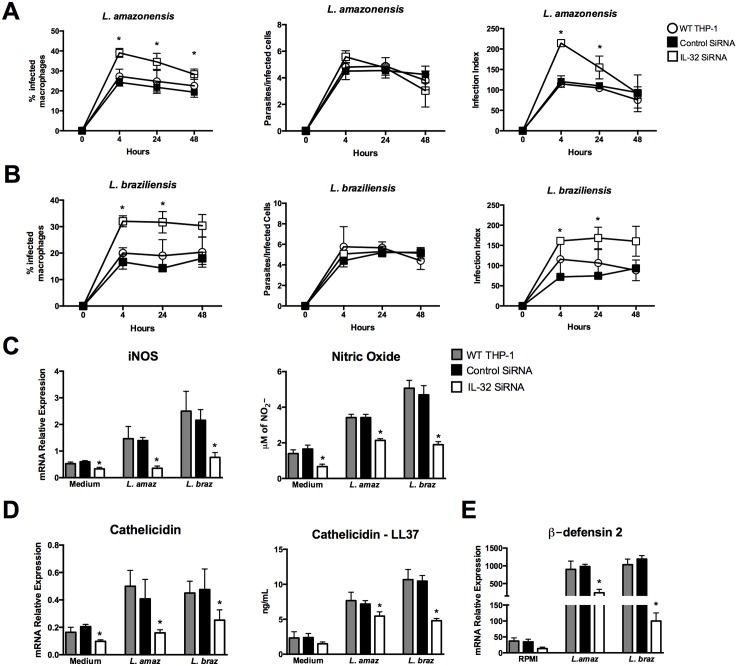
Silencing of IL-32 in human THP-1-derived macrophages increases *Leishmania* species infection. PMA-differentiated THP-1 cells (2.5 x 10^6^ cells/800 μL) were electroporated by using Amaxa Nucleofector Technology with IL-32 siRNA (for knockdown IL-32) and control siRNA according with protocol described by [38]. The final concentration of cells per well were 3 x 10^5^ cells/100 μL. After 24 h of transfection, cells were infected with promastigotes forms (1.5 x 10^6^ parasites) in the growth stationary phase of *L*. (*L*.) *amazonensis* (*L*. *amaz*), metacyclic promastigote forms (1.5 x 10^6^ parasites) of *L*. (*V*.) *braziliensis* (*L*. *braz*). After 4 h, cells were washed to remove non-internalized parasites and incubated for 24 h or 48 h. Cells were stained and percentage of infected macrophages, number of parasites per infected cells, and infection index were evaluated. (A) infection with *L*. *amazonensis*; (B) infection with *L*. *braziliensis*. iNOS (C—left panel), cathelidicin (D—left panel) and β-defensin 2 (E) mRNA expression were determined by quantitative real-time PCR (24 h). Production of nitrite (C—right panel) and LL-37 (D—right panel) was determined by Griess reagent and ELISA in supernatants, respectively (24 h). Values are expressed as means ± SEM of three independent experiments. *p < 0.05 (Control SiRNA vs IL-32 SiRNA).

To examine if endogenous IL-32γ controls *Leishmania* species infection, IL-32 was silenced in THP-1-derived macrophages prior to infection. To verify whether the transfection procedure may interfere with the capacity of THP-1-derived macrophages uptake of *Leishmania*, cells without transfection (WT THP-1) were also investigated. A significant increase in the percentage of macrophages infected with either *L*. (*L*.) *amazonensis* or *L*. (*V*.) *braziliensis* was observed after 4 h, 24 h and 48 h in the absence of IL-32 (Fig 5A and 5B). However, no differences were observed in the number of parasites per infected cell (Fig 5A and 5B). The infection index with both *Leishmania* species (4 h; 24 h) was increased in IL-32 knockdown cells (Fig 5A and 5B). Because silencing of IL-32 is a transitory process, we checked for IL-32 expression 48 h after infection. Indeed IL-32 silencing was reversed at this time (S4 Fig), potentially explaining the results at this time point of infection.

Fig 5C shows a significant decrease of iNOS and nitrite production after 24 h of infection with both *Leishmania* species in cells silenced for IL-32 compared to control cells. In addition, cathelicidin and β-defensin 2 mRNA expression as well as antimicrobial peptide LL-37 concentration were strongly reduced in THP-1-derived macrophages depleted of IL-32 (Fig 5D and 5E).

**Fig 6:**
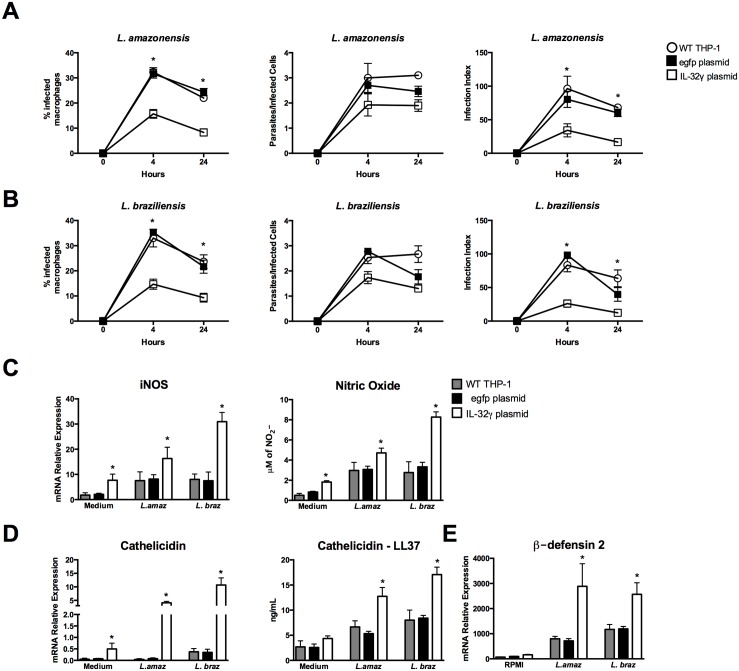
Decreased *Leishmania* species infection and increased leishmanicidal molecules after IL-32 overexpression in human THP-1-derived macrophages. PMA-differentiated THP-1 cells (2.5 x 10^6^ cells/800 μL) were electroporated by using Amaxa Nucleofector Technology with IL-32 plasmid (for IL-32 overexpression) and egfp plasmid (as a control). The final concentration of cells per well were 3 x 10^5^ cells/100 μL. After 24 h of transfection, cells were infected with promastigote forms (1.5 x 10^6^ parasites) in the growth stationary phase of *L*. (*L*.) *amazonensis* (*L*. *amaz*) or metacyclic promastigote forms (1.5 x 10^6^ parasites) of *L*. (*V*.) *braziliensis* (*L*. *braz*). After 4 h, non-internalized parasites were washed out and cells were incubated for 24 h or 48 h. Cells were stained and percentage of infected macrophages, number of parasites per infected cells, and infection index were evaluated. (A) infection with *L*. *amazonensis*; (B) infection with *L*. *braziliensis*. iNOS (C—left panel) and cathelidicin (D—left panel) and β-defensin 2 (E) mRNA expression were determined by quantitative real-time PCR (24 h). Nitrite production (C—right panel) and LL-37 production (D—right panel) were determined by Griess reagent and ELISA in supernatants, respectively (24 h). Values are expressed as means ± SEM of three independent experiments. *p < 0.05 (egpf plasmid vs IL-32γ plasmid).

In IL-32γ overexpressing cells, we observed a significant decrease in the percentage of infected cells and infection index after 4 h and 24 h with both *L*. (*L*.) *amazonensis and L*. (*V*.) *braziliensis* (Fig 6A and 6B). No differences were observed in the number of parasites per *L*. (*L*.) *amazonensis*- or L. (V.) braziliensis-infected cells (Fig 6B). In contrast, a reduction in the number of *L*. (*V*.) *braziliensis* parasites per infected cell after 4 h of infection was detected in IL-32γ-overexpressing cells (Fig 6B). In parallel, we observed a significant increase of iNOS mRNA expression and nitrite production after 24 h of infection with both *Leishmania* species in IL-32γ-transfected cells (Fig 6C). Moreover cathelicidin and β-defensin 2 mRNA expression as well as LL-37 peptide production were strongly increased when IL-32γ was overexpressed, especially in infection with *L*. (*V*.) *braziliensis* (Fig 6D and 6E).

## Discussion

The present study demonstrates the important role that endogenous IL-32γ plays in regulating cytokines and microbicidal molecules induced by *L*. (*L*.) *amazonensis* or *L*. (*V*.) *braziliensis* in THP-1-derived macrophages. Only IL-32γ mRNA was induced by parasites while splicing of IL-32γ into IL-32β and IL-32α was observed after LPS stimulation. In fact, these data are in accordance with previous results for LPS [7,42] and with our study showing that only IL-32γ can be detected in cutaneous and mucosal lesions of patients infected with *L*. (*Viannia*) species and in PBMCs cultured with *L*. (*V*.) *braziliensis* amastigotes [17].

Here, results indicate that *L*. (*L*.) *amazonensis* induces higher levels of IL-32γ mRNA and IL-32 protein than *L*. *(V*.*) braziliensis*. IL-32 is intracellularly expressed, as detected in cell lysates and confocal microscopy. It has been demonstrated that IL-32β can be driven towards cell membrane in U937 human monocytic cell line during cell activation [43]. In our hands, no specific IL-32 cell localization was detected in THP-1-derived macrophages during *Leishmania* species infection and it is not localized to parasite vacuoles in order to exert its biological functions.

In this study, TNFα production was higher in macrophages infected with *L*. (*L*.) *amazonenis* than *L*. (*V*.) *braziliensis*. This result could not be ascribed to LPS contamination (S5 Fig). Further, production of chemokine IL-8 was increased by both *Leishmania* species. We were unable to detect IL-1β production in *L*. (*L*.) *amazonenis*-infected THP-1 macrophages. In accordance, Shio *et al*. [44] demonstrated that *L*. (*L*.) *mexicana*, another species belonging to the same subgenus of *L*. (*L*.) *amazonensis*, has the ability to inhibit NLRP3 inflammasome activation and subsequently reduces IL-1β secretion in PMA-differentiated THP-1 cells. In addition, in mouse macrophages IL-1β is not induced by *L*. (*L*.) *amazonensis*; however, *in vivo* IL-1β is important to murine infection control [29]. In contrast to *L*. (*L*.) *amazonensis*, the present study showed that besides TNFα induction, *L*. (*V*.) *braziliensis* induced IL-1β and induced lower levels of IL-1Ra and IL-10 than *L*. (*L*.) *amazonensis*. This suggested a trend to more inflammatory profile in *L*. (*V*.) *braziliensis* than in *L*. (*L*.) *amazonensis* infection. The production of higher amounts of IL-1Ra and IL-10 in *L*. (*L*.) *amazonensis*-infected macrophages could contribute to balancing the inflammatory process. It is known that macrophage has the ability to produce inflammatory cytokines that is accompanied by an anti-inflammatory cytokine profile contributing to homeostasis of the immune response [45]. In the case of *Leishmania* infection the suppression of immune response can also lead to less inflammatory lesions or even to an anergic state in patients with severe cases of DCL caused by *L*. (*L*.) *amazonensis*. Less inflammatory properties of *L*. (*L*.) *amazonensis* in comparison with *L*. (*V*.) *braziliensis* have been described in mice [46] and human beings [21]. Thus, although in in vitro model presented here, *L*. (*L*.) *amazonensis* induced higher levels of IL-32γ and TNFα than *L*. (*V*.) *braziliensis* in human macrophages, the higher production of IL-10 and IL-1Ra induced by *L*. (*L*.) *amazonensis* can be responsible by further control of inflammatory process and immunosuppression in *in vivo* infections caused by this species. Nevertheless, we did not perform experiments to evaluate whether IL-10 or IL-1Ra can suppress the production of IL-32γ or TNFα induced by *Leishmania*. In fact, there is no report about the control of IL-32 production by anti-inflammatory cytokines. This point remains to be investigated.

One important point to be addressed is that, as reported above [21,47,48], it has been described that *L*. *(V*.*) braziliensis* tends to lead a T-cell hypersensitivity pole (strong production of IFNγ and TNFα) in patients with ML whereas *L*. (*L*.) *amazonensis* can lead to a T-cell hyposensitivity pole in patients with diffuse cutaneous leishmaniasis. However, both species can cause localized cutaneous leishmaniasis without clinical differences in lesions or immune responses [21] and there is no report about simultaneous comparison between cytokine productions by human macrophages infected with these two species. In human monocytes/macrophages from healthy donors the TNFα production after stimulation with *L*. (*V*.) *braziliensis* is low unless IFNγ has been added [49,50]. In mouse models, *L*. (*L*.) *braziliensis* is more inflammatory than *L*. (*L*.) *amazonensis* [47,51], but lesions caused by this latter species are bigger than those caused by *L*. (*V*.) *braziliensis* [46,52]. In addition, in mice *L*. *(V*.*) braziliensis* did not cause ML. Thus, it could be a surprise that *L*. (*L*.) *amazonensis* is inducing higher production of IL-32γ and TNFα in human THP-1 macrophages than *L*. (*V*.) *braziliensis*, but this could depend on the macrophage origin or activation status. It is noteworthy that human beings have IL-32 while mice lack this gene what can generate different responses when comparing macrophages from humans or mice.

Previously, we had demonstrated [53] that TNFα is a potent inducer of IL-32 in human synovial fibroblasts. Indeed, we now show that IL-32 protein production induced by *Leishmania* species and by LPS is also dependent on TNFα. On the other hand, IL-32γ upregulates the production of proinflammatory cytokines such as TNFα, IL-1β and IL-8 [3]. We then reasoned that IL-32γ could be responsible for cytokine regulation during *Leishmania* sp infection. Heinhuis *et al*. [41] demonstrated that IL-32γ is involved in cell death processes, and for this reason the influence of overexpression of IL-32 in THP-1 cells was evaluated only for 24 h, since after this time point, the increase of IL-32 inside the cells can lead to the cell death. After infection with both *Leishmania* species, TNFα and IL-8 were down regulated in THP-1 cells depleted of IL-32γ while overexpression of IL-32γ caused a strong increase in the production of these cytokines. The high levels of TNFα induced after IL-32γ overexpression in *L*. (*L*.) *amazonensis* infection are particularly noteworthy. Data from silencing (no effects on mRNA TNFα levels after 4 h) and from overexpression of IL-32 (increase of mRNA TNFα) suggest that the amount of IL-32γ can be a critical factor to increase transcription of TNFα. TNFα induction capacity was one of the first properties described for IL-32 [4]. Moreover, IL-32γ overexpression in THP-1 cells causes an increase in TNFα, IL-6 and IL-8 production [53] in accordance with our results. Heinhuis *et al*. [53] demonstrated that in cells overexpressing intracellular IL-32γ there is an enhanced TNFα mRNA stability, which explains higher TNFα levels than in control cells. In fact, IL-32γ seems to be required to control TNFα mRNA stability since in our hands silencing of IL-32γ decreased TNFα mRNA (24 h) whereas after overexpression of IL-32γ both TNFα mRNA (4 h) and TNFα protein were increased (24 h) in comparison to control cells. These data further suggest that IL-32γ can influence post-transcriptional mechanisms to increase TNFα during *Leishmania* species infection. We have previously reported increased expression of TNFα in lesions of ML patients infected with *L*. (*Viannia*) species and described a positive correlation between levels of TNFα and IL-32 [17], which is in agreement with these current data.

*L*. *(V*.*) braziliensis*-induced IL-1β and IL-1Ra as well as IL-10 induced by both *Leishmania* species were not affected by the up or down regulation of IL-32γ. That IL-32γ had no effect on IL-1β production was unexpected since silencing of IL-32 in THP-1 cells reduced TNFα, IL-8 and IL-1β after infection with MTB [6]. The negative regulator of IL-1β, IL-1Ra was induced by *L*. *(V*.*) brazilienis*, however this induction was not affected by IL-32γ levels, thus suggesting that IL-32γ is dispensable for IL-1β production but can enhance the effects of IL-1β during *L*. *(V*.*) braziliensis* infection. By contrast, *L*. *(L*.*) amazonensis* does not induce IL-1β and the induction of IL-1Ra by this parasite species was upregulated by IL-32γ, suggesting that if IL-1β is induced in vivo it can be controlled by IL-1Ra in an IL-32γ-dependent manner. In accordance, the induction of IL-1Ra by IL-32γ was described in PBMCs [45] and our data suggest that *Leishmania* parasites can partially subvert IL-32γ pro-inflammatory property by increasing IL-1Ra.

Infected THP-1-derived macrophages inhibit *Leishmania* species growth but do not eliminate them, at least until 48 h of culture. Cells depleted of IL-32 exhibited an increase in macrophage infection index after *Leishmania* species infection, which was reversed when IL-32γ was overexpressed. These effects were related to alterations in the percentage of infected cells and not to the number of parasites per cell. These results suggested that IL-32 is important to control infections caused by *L*. *(L*.*) amazonensis* and *L*. *(V*.*) braziliensis* in human macrophages. Therefore, we evaluated microbicidal molecules known to be involved in *Leishmania* control [30,54,55] as possible targets of IL-32γ. Our data demonstrate that IL-32γ is linked with the induction of iNOS, cathelicidin and β-defensin 2 expression and consequently NO, LL-37 peptide, and β-defensin 2 release during *Leishmania* species infection. These molecules were induced by IL-32 in influenza virus [48] and MTB infection [8]. NO is a classical leishmanicidal molecule in mouse macrophages [30] but human macrophages produce low levels of NO. Nevertheless, in some reports this was enough to contribute for *Leishmania* killing [56–58]. Cathelicidin plays a role in the control of lesions caused by *L*. *(L*.*) amazonensis* and prevents parasite dissemination in mice [59]. Thus, while in mice NO and cathelicidin are important molecules for anti-*Leishmania* macrophage activity in the absence of IL-32, in human cells anti-microbial peptides dependent on IL-32γ can contribute to control *Leishmania* infection.

In our experiments, low levels of microbicidal molecules were detected in uninfected macrophages, which decreased after IL-32 silencing (Fig 5). These results suggested that PMA used to differentiate THP-1 cells into macrophages can induce low levels of IL-32 that, in turn, contribute to induction of iNOS/NO and antimicrobial peptides. In fact, PMA can induce iNOS [60] and IL-32 [36] in THP-1 cells, thus both can contribute to increase *Leishmania*-induced microbicidal molecules.

Cathelicidin induction could be related to the IL-1Ra production, as described by Choi *et al*. [61]. They reported that LL-37 or IL-32γ enhanced human macrophage IL-1Ra production and subsequently led to the suppression of proinflammatory cytokines induced by IL-32γ as a possible negative feedback mechanism. According to our data, since IL-32γ is linked to IL-1Ra and LL-37 production after *L*. *(L*.*) amazonensis* infection, we suggest that IL-1Ra may indeed play a role in the balance of the inflammatory state caused by *L*. *amazonensis* suggesting a mechanism of feedback dependent on IL-32γ/LL-37/IL-1Ra.

In addition to the role of IL-32 in the production of microbicidal molecules we observed that after 4 h of infection the percentage of infected macrophages was inversely associated with IL-32 expression (Figs 5 and 6). As this is a short time for parasite proliferation, data suggest that IL-32 can modulate the uptake of the parasites. As it was shown before that IL-32 can induce differentiation of monocytes into macrophages increasing the phagocytosis capacity [62], we are now investigating whether IL-32 can control the phagocytosis process of *Leishmania* sp.

Our study was focused in understanding the role of IL-32 in innate immune response by evaluating human macrophage functions. However it is also known that IL-32 can drive the acquired immune response by inducing the differentiation of monocytes into dendritic cells. IL-32-matured and activated dendritic cells induce T helper (Th) lymphocyte differentiation into Th1 and Th17 cells [63,64], which are important cells to control leishmaniasis [65]. The role of IL-32-matured and activated dendritic cells must be further investigated in the context of *Leishmania* sp infections.

In summary, we demonstrate that *Leishmania* species induce IL-32γ, and suggest that these parasites can inhibit the IL-32γ splicing into the less pro-inflammatory isoforms IL-32β and IL-32α. Furthermore, we demonstrated that during *L*. *(V*.*) braziliensis* and *L*. *(L*.*) amazonensis* infection IL-32γ was differentially associated with the production of pro and anti-inflammatory mediators. In addition, IL-32γ upregulates the induction of microbicidal molecules, which may contribute to control *Leishmania* species infections (S6 Fig). The results suggest that IL-32γ is a crucial intracellular cytokine for the regulation of macrophage functions during *Leishmania* species infection that can result in different consequences of clinical manifestations of leishmaniasis caused by *L*. *(V*.*) braziliensis* and *L*. *(L*.*) amazonensis*. Next steps will include primary human macrophages to better understand the role of IL-32 in human leishmaniasis. Our current knowledge concerning the role of IL-32 in ATL might be useful and contribute to the development of new therapies.

## Supporting information

S1 Table ON-TARGETplus human IL-32 siRNA SMARTpool sequence. (PDF)

S2 Table Primers sequence. (PDF)

S1 Fig PMA-differentiated THP-1 cells (1x10^6^ cells/mL) were infected with promastigote forms (5x10^6^ parasites) in stationary phase of growth of *L*. (*L*.) *amazonensis* (*L*. *amaz*), metacyclic promastigote forms (5x10^6^ parasites) of *L*. (*V*.) *braziliensis* (*L*. *braz*) or LPS (100 ng/mL) as a positive control during 4 h. Non-internalized parasites were washed out and cells were incubated for 24 h or 48 h. (A) Time course production of IL-32γ after 4 h, 24 h and 48 h (left panel); Distribution of mRNA expression of isoforms of IL-32 after 24 h (right panel); determined by quantitative real-time PCR. (B) TNFα cytokine production in 4 h-culture supernatant, by ELISA. (C) LDH levels were determined by Cytotox 96 assay in supernatants after 24 h. Values are expressed as means ± SEM of three independent experiments. *p < 0.05 (Medium vs LPS, *L*. *amaz*, *L*. *braz*).
(TIF)

S2 Fig Time course of cytokine production induced by *L*. *amazonensis* and *L*. *braziliensis* in PMA-differentiated THP-1 cells. PMA-differentiated THP-1 cells (2 x 10^5^ cells/well) were infected with promastigote forms (1 x 10^6^ parasites) in the growth stationary phase of *L*. *(L*.*) amazonensis* (*L*. *amaz*) and metacyclic promastigote forms (1 x 10^6^ parasites) of *L*. *(V*.*) braziliensis* (*L*. *braz*) during 4 h. Cells were washed to remove non-internalized parasites and incubated for 24 h or 48 h. TNFα, IL-8, IL-1β, IL-1Ra and IL-10 concentrations were determined by ELISA in culture supernatants after 4 h, 24 h and 48 h of incubation. Values are expressed as means ± SEM of three independent experiments.
(TIF)

S3 Fig PMA-differentiated THP-1 cells (2.5x10^6^ cells/800 μL) were electroporated by using Amaxa Nucleofector Technology with IL-32 siRNA (for IL-32 knockdown) and control siRNA and IL-32 plasmid (for IL-32 overexpression) and egfp plasmid (as a control) according with protocol described in [28]. The final concentration of cells per well were 3 x 10^5^ cells/100 μL. After 24 h of transfection, cells were infected with promastigote forms (1.5x10^6^ parasites) in growth stationary phase of *L*. (*L*.) *amazonensis* (*L*. *amaz*) or metacyclic promastigote forms (1.5 x 10^6^ parasites) of *L*. (*V*.) *braziliensis* (*L*. *braz*). Afterwards, non-internalized parasites were washed out and cells were incubated for 24 h. After 4 h (A and C) and 24 h (B and D) incubation, mRNA expression and protein levels of TNFα were determined by quantitative real-time PCR and ELISA, respectively. Values are expressed as means ± SEM of three independent experiments. *p < 0.05 (Control SiRNA vs IL-32 SiRNA); (egpf plasmid vs IL-32γ plasmid); #p < 0.05 (*L*. *amaz* vs *L*. *braz*).
(TIF)

S4 Fig PMA-differentiated THP-1 cells (2.5x10^6^ cells/800 μL) were electroporated by using Amaxa Nucleofector Technology with IL-32 siRNA (for IL-32 knockdown) and control siRNA according with protocol described in [38]. The final concentration of cells per well were 3 x 10^5^ cells/100 μL. After 24 h of transfection, cells were infected with promastigote forms (1.5 x 10^6^ parasites) in growth stationary phase of *L*. (*L*.) *amazonensis* (*L*. *amaz*) or metacyclic promastigote forms (1.5 x 10^6^ parasites) of *L*. (*V*.) *braziliensis* (*L*. *braz*). Afterwards, non-internalized parasites were washed out and cells were incubated for 48 h. mRNA expression of Il-32 all (left) and γ isoform of IL-32 (right) were determined by quantitative real-time PCR. Values are expressed as means ± SEM of three independent experiments.
(TIF)

S5 Fig Evaluation of LPS contamination in parasite cultures. PMA-differentiated THP-1 cells (2 x 10^5^ cells/mL) were treated with polymyxin B (5 μg/mL) and infected with promastigote forms (1 x 10^6^ parasites) in the growth stationary phase and metacyclic promastigote forms (1 x 10^6^ parasites) of *L*. *(V*.*) braziliensis* (*L*. *braz*). After 4 h supernatants were collected and cells were washed to remove non-internalized parasites and incubated for 24, 48 h in the presence of polymyxin B. TNFα protein levels were determined by ELISA in supernatants. Values are expressed as means ± SEM of three independent experiments. *p < 0.05 (Medium vs *L*. *amaz*, *L*. *braz*); #p < 0.05 (*L*. *amaz* vs *L*. *braz*).
(TIF)

S6 Fig An overview of cytokines and microbicidal molecules induced by *Leishmania* species in PMA-differentiated human THP-1 cells. IL-32γ and IL-8 are induced by *L*. (*L*.) *amazonensis* and *L*. (*V*.) *braziliensis* at similar levels. *L*. (*L*.) *amazonensis* induces higher levels of TNFα, IL-10 and IL-1Ra than *L*. (*V*.) *braziliensis*, which induces higher levels of IL-1β. TNFα and IL-8 production is mediated by IL-32γ in infections caused by both species whereas IL-1Ra production is only dependent on IL-32γ in *L*. (*L*.) *amazonensis* infection. In addition, *L*. (*V*.) *braziliensis*–induced IL-1β is not dependent on IL-32γ as well as production of IL-10 induced by both parasite species. Considering microbicidal molecules IL-32γ contributes similarly for their production in cells infected with both *Leishmania* species. The differential control of cytokines induced after *L*. (*L*.) *amazonensis* and *L*. (*V*.) *braziliensis* infections by IL-32γ can contribute for different clinical outcomes of disease caused by theses parasites.
(TIF)
