# Supplementary material for: Cytokines and microbicidal molecules regulated by IL-32 in THP-1-derived human macrophages infected with New World Leishmania species
Source: PLoS Negl Trop Dis. 2017 Feb 27;11(2):e0005413. doi: 10.1371/journal.pntd.0005413 (PMC5344527; doi:10.1371/journal.pntd.0005413)
Supplement: S1 Table — (PDF) [file pntd.0005413.s001.pdf]

Target sequence 1: AGACAGUGGCGGCUUAUUA

Target sequence 2: AGAAAGAGAUGGAUUACGG

Target sequence 3: CGAAGGUCCUCUCUGAUGA

Target sequence 4: GAGCUCACUCCUCUACUUG

Supplementary Table 1
ON-TARGETplus Human IL-32 siRNA SMARTpool sequence
