# Supplementary material for: Cytokines and microbicidal molecules regulated by IL-32 in THP-1-derived human macrophages infected with New World Leishmania species
Source: PLoS Negl Trop Dis. 2017 Feb 27;11(2):e0005413. doi: 10.1371/journal.pntd.0005413 (PMC5344527; doi:10.1371/journal.pntd.0005413)
Supplement: S2 Table — (PDF) [file pntd.0005413.s002.pdf]

GAPDH FW primer: 5'-AGG-GGA-GAT-TCA-GTG-TGG-TG-3'

GAPDH RV primer: 5'-CGA-CCA-CTT-TGT-CAA-GCT-CA-3'

IL-32all FW primer: 5'-AGGACGTGGACAGGTGATGTC-3'

IL-32all RV primer: 5'-GTCTCCAGGTAGCCCTCTTTGA-3'

IL-32γ FW primer: 5'-AGGCCCGAATGGTAATGCT-3'

IL-32γ RV primer: 5'-CCACAGTGTCCTCAGTGTCACA-3'

TNF-α FW primer: 5'-CCTCTCTCTAATCAGCCCTCTG-3'

TNF-α RV primer: 5'-GAGGACCTGGGAGTAGATGAG-3'

IL-8 FW primer: 5'-ACTGAGAGTGATTGAGAGTGGAC-3'

IL-8 RV primer: 5'-AACCCTCTGCACCCAGTTTTC-3'

IL-1β FW primer: 5'-CAGCTACGAATCTCCGACCAC-3'

IL-1β RV primer: 5'-GGCAGGGAACCAGCATCTTC-3'

IL-1Ra FW primer: 5'-GCCTCCGCAGTCACCTAAT-3'

IL-1Ra RV primer: 5'-TCCCAGATTCTGAAGGCTTG-3'

IL-10 FW primer: 5'-CAACCTGCCTAACATGCTTCG-3'

IL-10 RV primer: 5'-TCATCTCAGACAAGGCTTGGC-3'

iNOS FW primer: 5'-GCGCAGACATGATCGCCATA-3'

iNOS RV primer: 5'-CCTCACCGAACTCACCAGC-3'

Cathelicidin FW primer: 5'-TGCCCAGGTCCTCAGCTAC-3'

Cathelicidin RV primer: 5'-GTGACTGCTGTGTCGTCCT-3'

β-defensin 2 FW primer: 5'-GGT GTT TTT GGT GGT ATA GGC G-3'
β-defensin 2 RV primer: 5'-AGG GCA AAA GAC TGG ATG ACA-3'

Supplementary Table 2
Primers sequence
