# Supplementary figures and images for: Cytokines and microbicidal molecules regulated by IL-32 in THP-1-derived human macrophages infected with New World Leishmania species
Source: PLoS Negl Trop Dis. 2017 Feb 27;11(2):e0005413. doi: 10.1371/journal.pntd.0005413 (PMC5344527; doi:10.1371/journal.pntd.0005413)

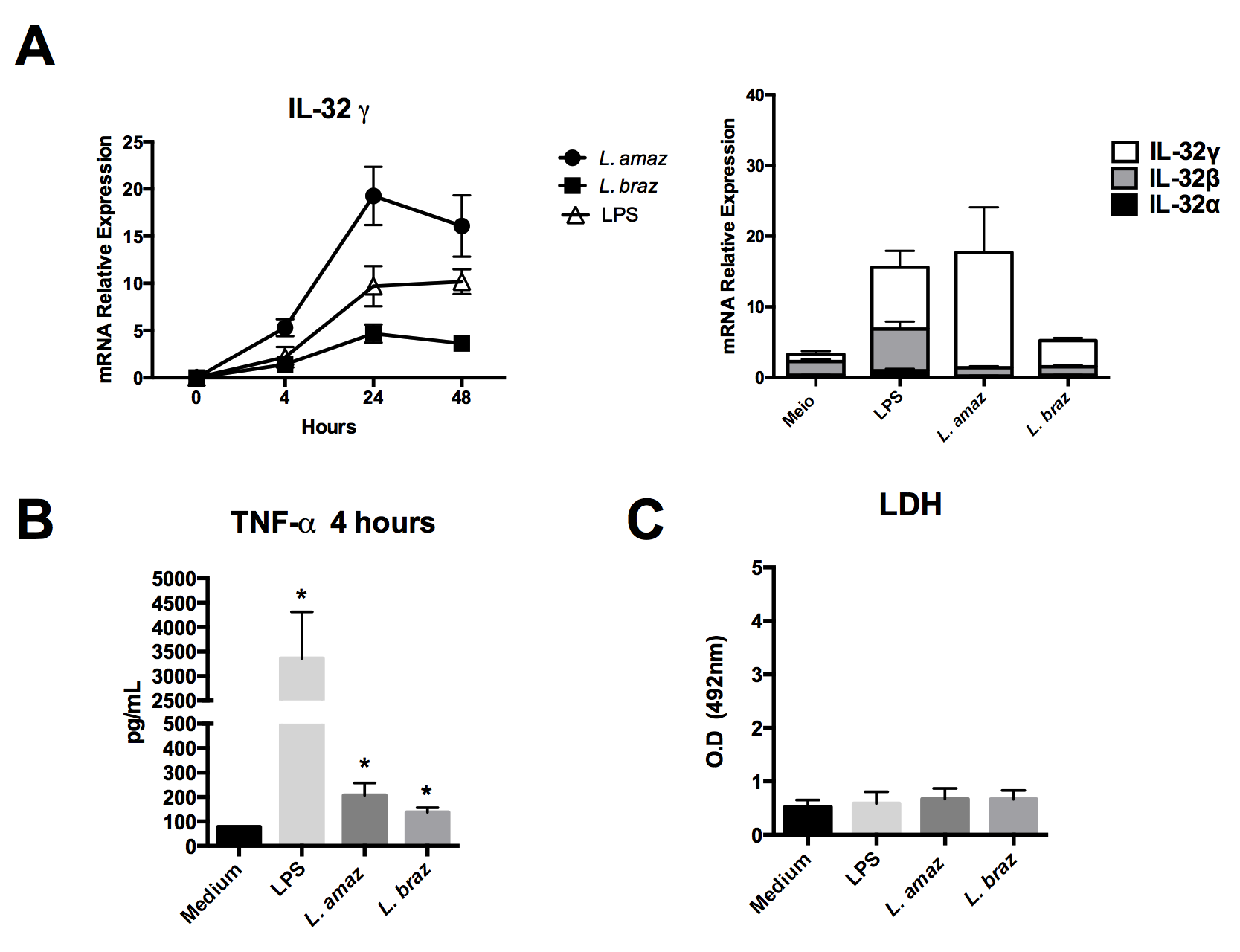

Supplement: S1 Fig — Non-internalized parasites were washed out and cells were incubated for 24 h or 48 h. (A) Time course production of IL-32γ after 4 h, 24 h and 48 h (left panel); Distribution of mRNA expression of isoforms of IL-32 after 24 h (right panel); determined by quantitative real-time PCR. (B) TNFα cytokine production in 4 h-culture supernatant, by ELISA. (C) LDH levels were determined by Cytotox 96 assay in supernatants after 24 h. Values are expressed as means ± SEM of three independent experiments. *p < 0.05 (Medium vs LPS, L. amaz, L. braz). (TIF) [file pntd.0005413.s003.tif]

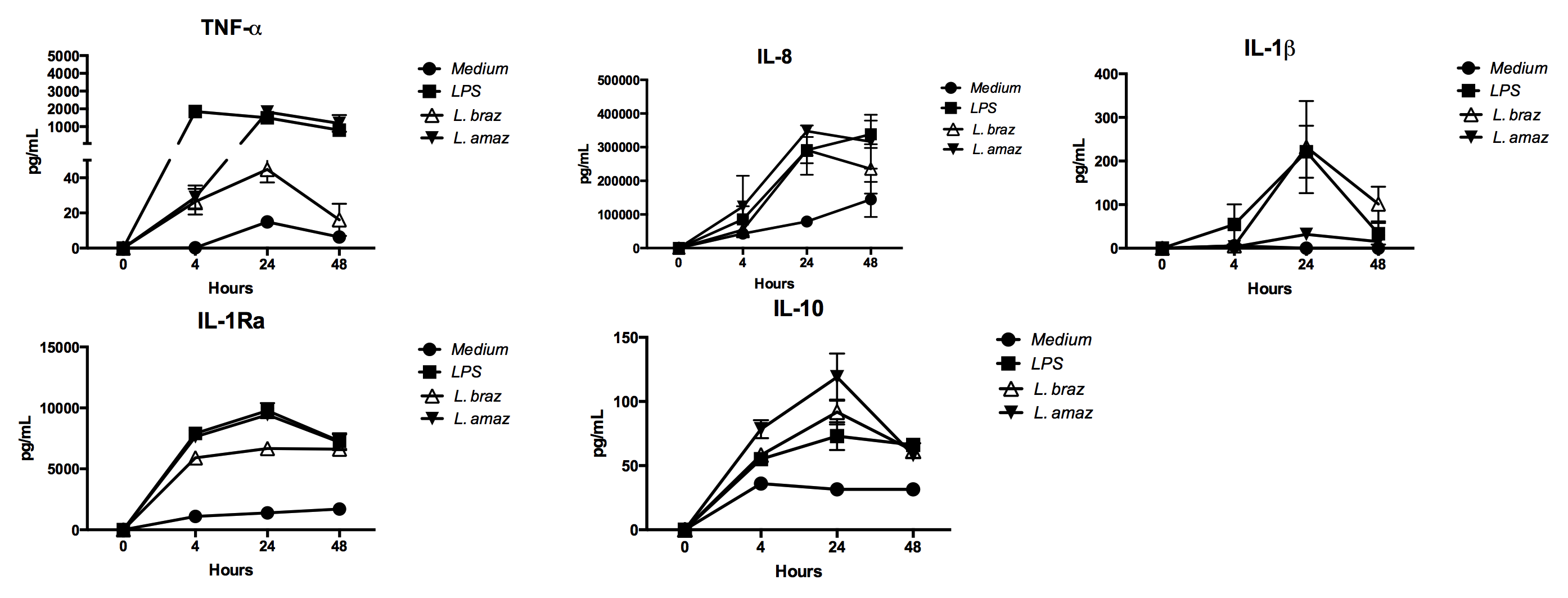

Supplement: S2 Fig — PMA-differentiated THP-1 cells (2 x 10^5^ cells/well) were infected with promastigote forms (1 x 10^6^ parasites) in the growth stationary phase of L. (L.) amazonensis (L. amaz) and metacyclic promastigote forms (1 x 10^6^ parasites) of L. (V.) braziliensis (L. braz) during 4 h. Cells were washed to remove non-internalized parasites and incubated for 24 h or 48 h. TNFα, IL-8, IL-1β, IL-1Ra and IL-10 concentrations were determined by ELISA in culture supernatants after 4 h, 24 h and 48 h of incubation. Values are expressed as means ± SEM of three independent experiments. (TIF) [file pntd.0005413.s004.tif]

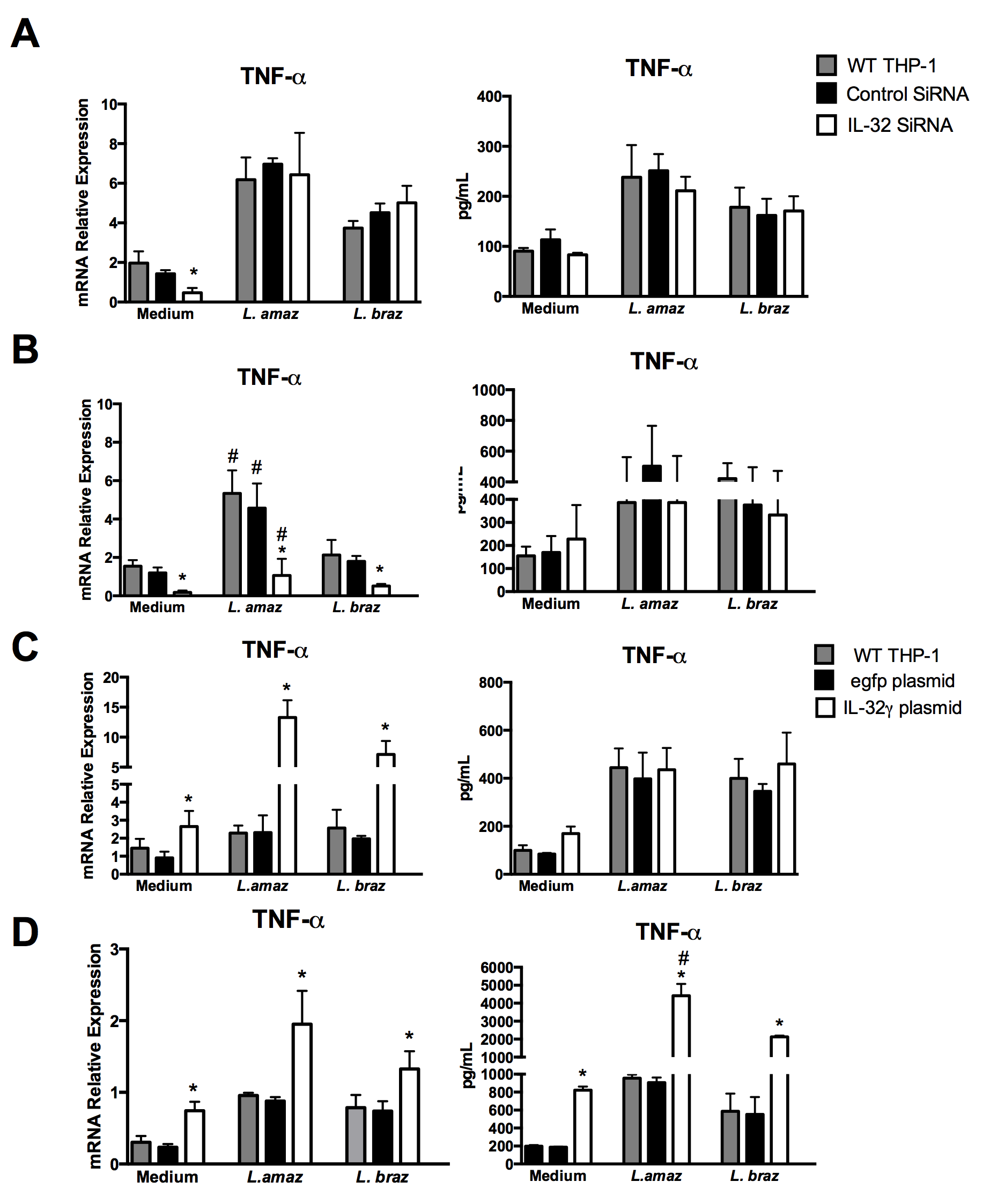

Supplement: S3 Fig — The final concentration of cells per well were 3 x 10^5^ cells/100 μL. After 24 h of transfection, cells were infected with promastigote forms (1.5x10^6^ parasites) in growth stationary phase of L. (L.) amazonensis (L. amaz) or metacyclic promastigote forms (1.5 x 10^6^ parasites) of L. (V.) braziliensis (L. braz). Afterwards, non-internalized parasites were washed out and cells were incubated for 24 h. After 4 h (A and C) and 24 h (B and D) incubation, mRNA expression and protein levels of TNFα were determined by quantitative real-time PCR and ELISA, respectively. Values are expressed as means ± SEM of three independent experiments. *p < 0.05 (Control SiRNA vs IL-32 SiRNA); (egpf plasmid vs IL-32γ plasmid); #p < 0.05 (L. amaz vs L. braz). (TIF) [file pntd.0005413.s005.tif]

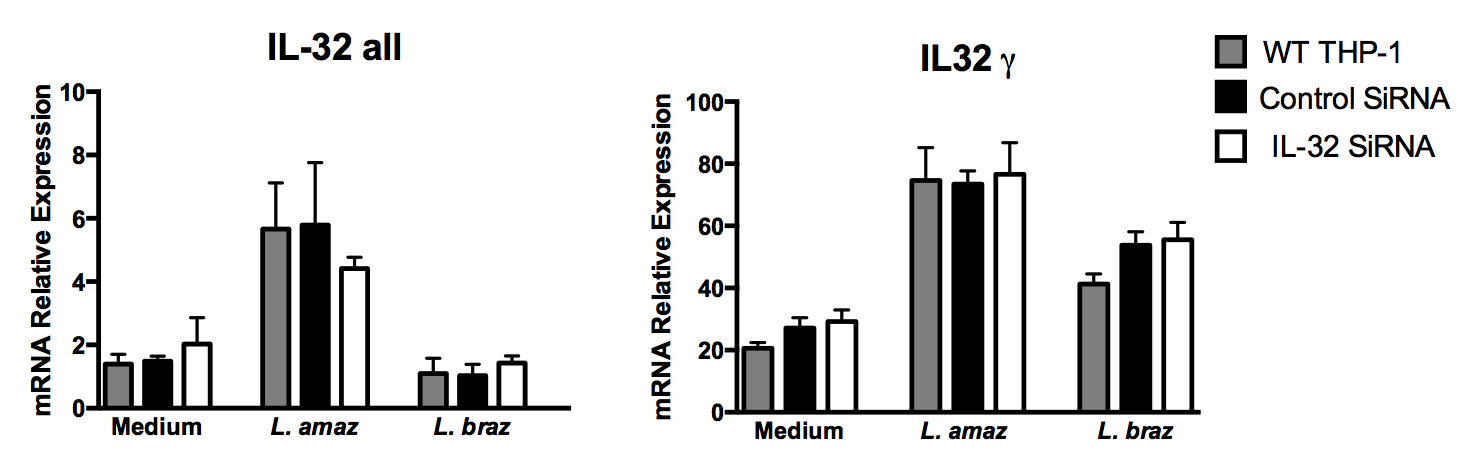

Supplement: S4 Fig — The final concentration of cells per well were 3 x 10^5^ cells/100 μL. After 24 h of transfection, cells were infected with promastigote forms (1.5 x 10^6^ parasites) in growth stationary phase of L. (L.) amazonensis (L. amaz) or metacyclic promastigote forms (1.5 x 10^6^ parasites) of L. (V.) braziliensis (L. braz). Afterwards, non-internalized parasites were washed out and cells were incubated for 48 h. mRNA expression of Il-32 all (left) and γ isoform of IL-32 (right) were determined by quantitative real-time PCR. Values are expressed as means ± SEM of three independent experiments. (TIF) [file pntd.0005413.s006.tif]

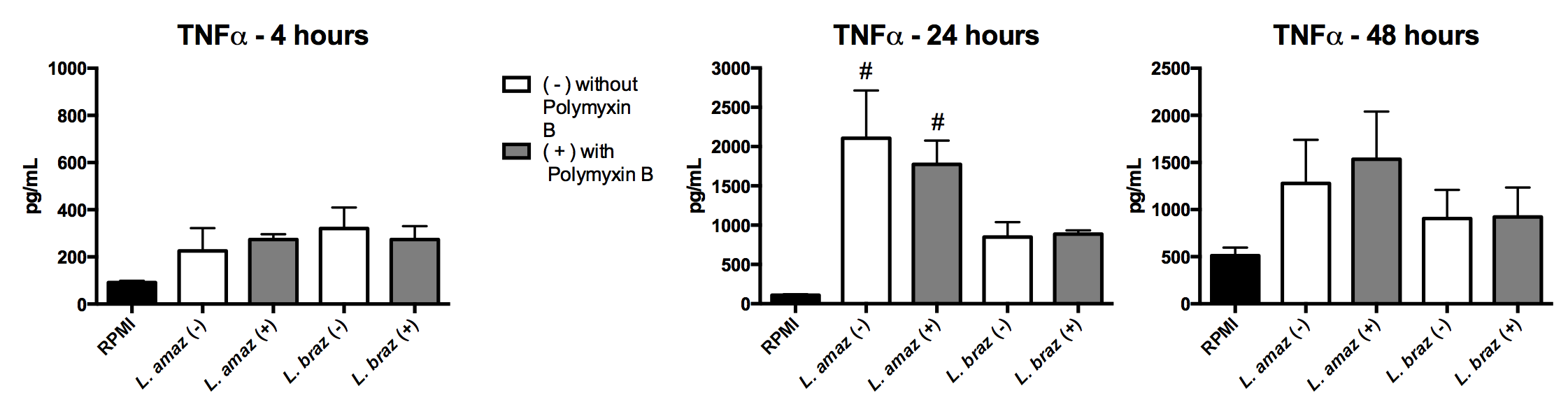

Supplement: S5 Fig — PMA-differentiated THP-1 cells (2 x 10^5^ cells/mL) were treated with polymyxin B (5 μg/mL) and infected with promastigote forms (1 x 10^6^ parasites) in the growth stationary phase and metacyclic promastigote forms (1 x 10^6^ parasites) of L. (V.) braziliensis (L. braz). After 4 h supernatants were collected and cells were washed to remove non-internalized parasites and incubated for 24, 48 h in the presence of polymyxin B. TNFα protein levels were determined by ELISA in supernatants. Values are expressed as means ± SEM of three independent experiments. *p < 0.05 (Medium vs L. amaz, L. braz); #p < 0.05 (L. amaz vs L. braz). (TIF) [file pntd.0005413.s007.tif]

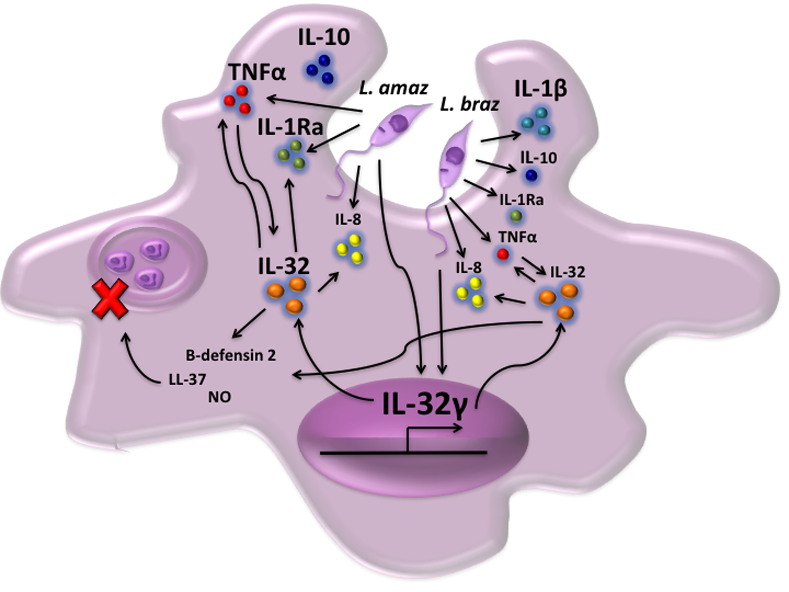

Supplement: S6 Fig — IL-32γ and IL-8 are induced by L. (L.) amazonensis and L. (V.) braziliensis at similar levels. L. (L.) amazonensis induces higher levels of TNFα, IL-10 and IL-1Ra than L. (V.) braziliensis, which induces higher levels of IL-1β. TNFα and IL-8 production is mediated by IL-32γ in infections caused by both species whereas IL-1Ra production is only dependent on IL-32γ in L. (L.) amazonensis infection. In addition, L. (V.) braziliensis–induced IL-1β is not dependent on IL-32γ as well as production of IL-10 induced by both parasite species. Considering microbicidal molecules IL-32γ contributes similarly for their production in cells infected with both Leishmania species. The differential control of cytokines induced after L. (L.) amazonensis and L. (V.) braziliensis infections by IL-32γ can contribute for different clinical outcomes of disease caused by theses parasites. (TIF) [file pntd.0005413.s008.tif]
